# Location angle of second mesio-buccal canal in maxillary molars of an Indian population: an in vivo retrospective CBCT evaluation and proposal of a new classification

**DOI:** 10.7717/peerj.14234

**Published:** 2022-10-10

**Authors:** Kishor Vhorkate, Kulvinder Banga, Ajinkya M. Pawar, Shugufta Mir, Suraj Arora, Dian Agustin Wahjuningrum, Anuj Bhardwaj, Alexander Maniangat Luke

**Affiliations:** 1 Department of Conservative Dentistry and Endodontics, Nair Hospital Dental College, Mumbai, Maharashta, India; 2 Department of Restorative Dental Sciences, College of Dentistry, King Khalid University, Abha, Saudi Arabia; 3 Department of Conservative Dentistry, Universitas Airlingga, Faculty of Dental Medicine, Surabaya City, East Java, Indonesia; 4 Department of Conservative Dentistry and Endodontics, College of Dental Sciences & Hospital, Rau, Indore, India; 5 Department of Clinical Science, College of Dentistry, Ajman University, Al-Jurf, Ajman, United Arab Emirates; 6 Center of Medical and Bio-allied Health Sciences Research, Ajman University, Al-Jurf, Ajman, United Arab Emirates

**Keywords:** Angular classification, Banga Vhorkate and Pawar’s classification, CBCT, Endodontics, Maxillary molars, Mesio-buccal root

## Abstract

**Background:**

The current investigation was designed for predicting the location angle of second mesio-buccal root canal in permanent maxillary (first and second) molars with the aid of proposed measuring points and line using cone beam computed tomography in an Indian population.

**Methods:**

Three-hundred and twenty-four scans of permanent maxillary (first (*n* = 162) and second (*n* = 162)) molars with mesio-buccal 2 root canals and unassociated to the current evaluation were acquired. The maxillary molars were viewed with CSI imaging software. The images were captured and were further assessed using 3D Slicer. The assessment included of measuring the distance between the main mesio-buccal and mesio-buccal 2 canal and the angle at which the MB2 it is located utilizing proposed lines joining the disto-buccal and palatal canals. The data was tabulated for the incidence of various angles where the MB2 is located and MB-MB2 distance was determined. The angles denoted were either positive; I (0.1° to 1.9°), II (2° to 4°), III (>4°) or negative I (−0.1° to −1.9°), II (−2° to −4°), III (>−4°). On the data tabulated a new Banga Vhorkate and Pawar’s (BVP’s) angular classification for maxillary molars was proposed.

**Results:**

The existence of positive angle III was found in 41.35% of maxillary first molars (36 right and 31 left of 162), whereas positive angle II appeared in 41.98% of maxillary second molars (32 right and 36 left of 162). The MB1–MB2 in maxillary 1^st^ molar is seen to be 3.12–3.31 mm and this distance in maxillary 2^nd^ molar is 2.8–3.1 mm. The disto-buccal to palatal canal orifice mean distance was 5.06–5.22 mm in maxillary first molars and 4.9–5.8 mm in maxillary second molars.

**Conclusion:**

Accurate diagnosis of the location of second mesio-buccal canal increases the success rate of endodontic treatment and a better prognosis. The new proposed classification may be considerably helpful in the urge to locate the mesio-buccal 2 canal.

## Introduction

The inability of clinicians to recognize the heterogeneity of root canal therapy and to locate every canal in the tooth is one of the main causative factors for the failure of the therapy (Lee et al., 2020). Understanding the anatomy of the tooth structure and the root canal system in depth is essential to the success of the treatment (Akbarzadeh et al., 2017). Thorough knowledge is obligatory because every tooth is unique anatomically, having a different number of canals and roots and variation in the presence of the location of the canals in maxillary molars (Kewalramani, Murthy & Gupta, 2019).

The literature reveals that, during the root canal treatment of the maxillary molars, locating all canals is often missed by clinicians (Kewalramani, Murthy & Gupta, 2019). They have a minimum of three roots (mesiobuccal, distobuccal and palatal roots) and a maximum of up to four roots (Cantatore, Berutti & Castellucci, 2006). However, studies have shown that one or two roots can occur in maxillary molars (Mashyakhy et al., 2019; Al Mheiri et al., 2020). There are even claims of these teeth comprising five roots (Barbizam, Ribeiro & Filho, 2004; Borghesi et al., 2019). Each root exhibits unique internal anatomical variations in terms of shape, form, and size. However, the mesiobuccal (MB) root is perpetually believed to present an anatomical and clinical challenge. This is in accordance with the retreatment cases observed by Cleghorn, Christie & Dong (2006), where approximately 93% of cases failed due to missed canals in MB roots. Furthermore, they affirmed the presence of two or more canals in the MB root in approximately 56.8% of cases.

There are various ways to study root canal anatomies (Naseri et al., 2018; Kewalramani, Murthy & Gupta, 2019; Mashyakhy et al., 2019; Al Mheiri et al., 2020). Laboratory studies employ a wide range of methods to study root canal anatomy, including decalcification with injection of India ink, Chinese ink, hematoxylin dye, plastic, or metal castings, *in vitro* endodontic access with radiography and instruments or instruments only, *in vitro* radiopaque gel infusion and radiography, *in vitro* root canal treatment (RCT), *in vitro* radiography, *in vitro* macroscopic examination, scanning electron microscope examination of pulp floor, grinding or sectioning and, most recently, cone-beam computed tomography and microcomputed tomography (Ahmed, 2022). The clinical methods encompass retrospective analysis of patient records, radiography of all teeth, *in vivo* radiographic examination, and clinical evaluation during endodontic treatment using magnification or a dental operating microscope (DOM) or during endodontic treatment where magnification was not specified (Cleghorn, Christie & Dong, 2006).

In the past few years, scientific advancement has resulted in a significant reduction in the dose of radiation generated by the latest-generation devices, allowing for increasingly high-resolution diagnostic exams and applications in every branch of dentistry. Furthermore, the possibility of using ionizing-radiation-free diagnostic exams in dentistry has led to scientific research in this area yielding interesting results that inspire confidence. Assessments of this method, in addition to providing useful diagnostic indicators, could be used with absolute credibility for patient follow-up because they can be repeated over relatively short distances without exacerbating biological damage (Reda et al., 2021).

It has been suggested that approximately 96.1% of maxillary molars indeed have a second mesiobuccal canal (MB2) in the American population (Blattner et al., 2010). In the Indian population, the distribution of MB2 canals in maxillary first molars ranges from 61.9% to 86.36% (Kewalramani, Murthy & Gupta, 2019; Shetty et al., 2017). The supporting *in vitro* and *in vivo* studies of MB canal variation are summarized in Tables 1 and 2. There has been competent literature on the incidence of a second mesiobuccal canal; however, lacunae exist regarding the actual location of this type of canal in any population. Most of the studies concentrate on the incidence of MB2 and/or the MB1–MB2 canal distance (Lee et al., 2020; Akbarzadeh et al., 2017; Cotton et al., 2007; Tuncer, Haznedaroglu & Sert, 2010; Huumonen et al., 2006) but rarely measure the angles between the canals, which may help to depict the actual location.

**Table 1:** *In vitro* studies evaluating the incidence of mesiobuccal 2 canal in maxillary molars of different populations.

Sr no	Author	Population	Evaluation method	Sample size	Incidence % of maxillary 1^st^ molar	Incidence % of maxillary 2^nd^ molar
1	Vertucci (1984)	Unknown	Clearing	100 – 1^st^ molar	51%	50%
				100 – 2^nd^ molar		
2	Pécora et al. (1992)	Brazilian	Clearing	120 – 1^st^ molar	25%	42%
				200 – 2^nd^ molar		
3	Rwenyonyi et al. (2007)	Ugandan	Clearing	221 – 1^st^ molar	95.9%	95.9%
				221 – 2^nd^ molar		
4	Alaçam et al. (2008)	Unknown	Clearing+operating microscope,	100 – 1^st^ molar	74%	–
5	Smadi & Khraisat (2007)	Jordanian	Magnifying dental loupes	100 – 1^st^ molar	77.32%	–
6	Degerness & Bowles (2010)	American	Sectioned+stereomicroscope	90 – 1^st^ molar	79.8	60.3
				63 – 2^nd^ molar		
7	Tuncer, Haznedaroglu & Sert (2010)	Turkish	Examination with dental loups	110 – 1^st^ molar	78%	–
8	Peeters, Suardita & Setijanto (2011)	Indonesian	Sectioning	308 – 1^st^ molar	68.5%	–
9	Vizzotto et al. (2013)	Brazilian	Radiographic examination (RE) and CBCT	89 – 1^st^ molar	67%	–
10	Singh & Pawar (2015)	Indian	Clearing	100 – 1^st^ molar	28%	18%
				100 – 2^nd^ molar		
11	Alrahabi & Sohail Zafar (2015)	Saudi Arabian	Clearing+CBCT	100 – 1^st^ molar	70.6%	–
12	Alfouzan et al. (2019)	Saudi Arabian	Radiographic	35 – 1^st^ molar	97%	93%
				30 – 2^nd^ molar		
13	Sahiti et al. (2020)	Indian	Radiographic	6,945 – 1^st^ molar	2,534	–
14	Camargo Dos Santos et al. (2020)	Brazilian	Micro-CT scanning and 3D reconstruction	96 – 1^st^ molar	43.9%	

**Table 2:** *In vivo* studies evaluating the incidence of mesiobuccal 2 canal in maxillary molars of different populations.

Sr no	Author	Population	Method of evaluation	Sample size	Incidence % of maxillary 1^st^ molar	Incidence % of maxillary 2^nd^ molar
1	Stropko (1999)	Unknown	Clinical observation	1,096 – 1^st^ molar	73.2%	50.7%
				611 – 2^nd^ molar		
2	Wolcott et al. (2002)	American	Clinical observation	1,873	61%	36%
3	Buhrley et al. (2002)	Unknown	Endodontic treatment+Dental Loupes	312	57.4 %	55.3%
4	Wolcott et al. (2005)	American	Endodontically treated and retreated	5,616	60%	35%
5	Plotino et al. (2013)	Unknown	CBCT	201	40.3%	15.1%
6	Silva et al. (2013)	Brazilian	CBCT	314 – 1^st^ molar	42.63%	34.32%
				306 – 2^nd^ molar		
7	Sujith et al. (2014)	Unknown	Clinical observation	60 – 1^st^ molar	70%	–
8	Hasan & Khan (2014)	Pakistani	Clinical observation	53 – 1^st^ molar	50.9%	–
9	Guo et al. (2014)	American	CBCT	317 – 1^st^ molar	65.6%	
10	Nikoloudaki, Kontogiannis & Kerezoudis (2015)	Greek	CBCT	273 – 1^st^ molar	53.41%	
11	Agwan et al. (2015)	Saudi Arabian	Endodontic treatment+Dental Loupes	100 – 1^st^ molar	45%	–
12	Betancourt et al. (2015)	Chilean	CBCT	225 – 2^nd^ molar	–	48%
13	Wu et al. (2017)	Chinese	CBCT	2,412 – 2^nd^ molar	–	68.09%
14	Olczak & Pawlicka (2017)	Polish	CBCT	185 – 1^st^ molar	59.5%	30.5
				207 – 2^nd^ molar		
15	Shetty et al. (2017)	Indian	CBCT	66 – 1^st^ molar	86.36%	29.4%
				34 – 2^nd^ molar		
16	Naseri et al. (2018)	Iranian	CBCT	157 – 2^nd^-molar	–	67.51%
17	Martins et al. (2018)	Worldwide analysis	CBCT	5,250 – 1^st^ molar	73.8%	-
18	Fernandes et al. (2019)	South African	CBCT	200 patient	92%	69%
				800 teeth		
19	Lee et al. (2020)	South Korean	CBCT	76 – 1^st^ molar	86.8%	28.9%
				135 – 2^nd^ molar		
20	Manigandan et al. (2020)	Indian	Clinical observation	122	93%	86%
21	Pawar et al. (2021)	Indian	CBCT	966 – 1^st^ molar	77.5%	

With the above background, the current study aimed to (i) assess the location of MB2 canals with respect to the disto-buccal canal and palatal canal in the maxillary molars, (ii) measure the angles and distances between the canals using CBCT images in an Indian population and (iii) devise and employ a classification based on information gleaned from an examination of the angles between the DB-palatal and MB1–MB2 lines.

## Materials and Methods

### Investigation design and ethical approval

The current retrospective study protocol received approval from the institutional ethical committee of the College of Dental Sciences and Hospital (CDSH/08/2022). The CBCT scans were collected for various diagnostic purposes, such as disimpaction, orthodontic treatment, implant treatment planning, and maxillofacial diagnosis. A total of 324 permanent maxillary (first and second) molars with mesiobuccal teeth having two root canals were acquired from the collection. The patients included in the study were 18–75 years old. Only teeth without caries, fillings, endodontic treatment, periapical lesions, and root resorption were included in the current study for evaluation. Given the retrospective nature of the study and the use of anonymous clinical data in the analysis, informed consent was not sought.

### Radiographic techniques

A CBCT scanner Kodak care stream 9,000 with the configuration of 80 kV, 5 mA and exposure of 10.8 s was set. The voxel image size was 0.2 mm with a slice thickness of 0.1 mm. The scans were obtained with minimum exposure not distorting the image quality. These scans (CS 3D Imaging Software) were taken and handled by a trained radiologist strictly following the manufacturer’s guidelines. The interpretation of scans was reproduced using CSI Imaging Software calibration. The Cohen kappa (k) coefficient was used to determine inter- and intraexaminer reliability. The interexaminers included two endodontists and one maxillofacial radiologist. At 10-day interval, intrarater reliability was evaluated. Both inter- and intraexaminer reliability were excellent, with a kappa value of 0.91.

### Image evaluation and data extraction

The maxillary molars were evaluated with CSI Imaging Software using coronal sections until the second mesiobuccal canal orifice was visible. Using 3D slicer software, the images captured were further assessed. The restructuring of images in three planes (sagittal, axial and coronal) thus obtains 3-D images. The images were gathered in a manner where the tooth’s longitudinal axis was perpendicular to the cementoenamel junction plane. The morphological presence of MB2 canals was easily determined in this manner. Upon reading and discovering the presence of the MB2 canal, a plane was set for different measurements. This plane was referenced from 1 mm inferior to the floor of pulp to the apex point of the tooth root. This analysis was repeated for other canals of the root (MB1, DB and P). The assessment included the distance between the main mesiobuccal and mesiobuccal 2 canals and the angle at which MB2 was located utilizing proposed lines joining the distobuccal and palatal canals by a single operator. Three consecutive measurements were performed by marking the lines three times in succession while measuring the angle, and the mean was tabulated and analyzed. Furthermore, to limit the single assessor’s optical fatigue, data acquisition was limited to examining 5–6 scans per day. The data were tabulated for the incidence of various angles where MB2 is located, and the MB1–MB2 distance was determined. Distinct analyses were performed for the maxillary first and second molars.

The readings and observations obtained were tabulated for further statistical evaluation. For this, SPSS (v 26.0; IBM, Armonk, NY, USA) was used to determine the results of the study. The intergroup data were evaluated using one-way ANOVA. The pairwise analysis was determined *via* a *post hoc* test.

### Proposal of a angular classification

The BVP’s classification was proposed on the basis of data obtained by the analysis of angles between the MB1–MB2 and DB-palatal lines (Table 3).

**Table 3:** Angles proposed by Banga, Vhorkate and Pawar’s (BVP’s) angular classification.

Angle classified in positive angle and negative angle	Angle in degree
Positive angle I	0.1° to 1.9°
Positive angle II	2° to 4°
Positive angle III	>4°
Negative angle I	−0.1° to −1.9°
Negative angle II	−2° to −4°
Negative angle III	>−4°

The proposed BVP’s classification was based on the following:

1. Line drawn from the center of the disto-buccal canal orifice (DB) to the center of the palatal canal orifice (P) (red line).
2. Equivocal line drawn from the center of the first mesiobuccal canal (MB1), which is collateral to the line connecting DB and P (dotted black line).
3. Line drawn from the center of the first mesiobuccal canal orifice (MB1) to the center of the second mesiobuccal canal orifice (MB2) (black line).
4. Angle measured between the equivocal line, which is collateral to the disto-buccal (DB) to palatal (P) line, and the line between the first mesio-buccal canal (MB1) and the second-mesio-buccal canal (MB2).
  - The angle is described as positive when the MB2 canal is more mesial than the equivocal line collateral to the line connecting the DB and P canals passing through MB1 (Fig. 1A).

**Figure 1:**
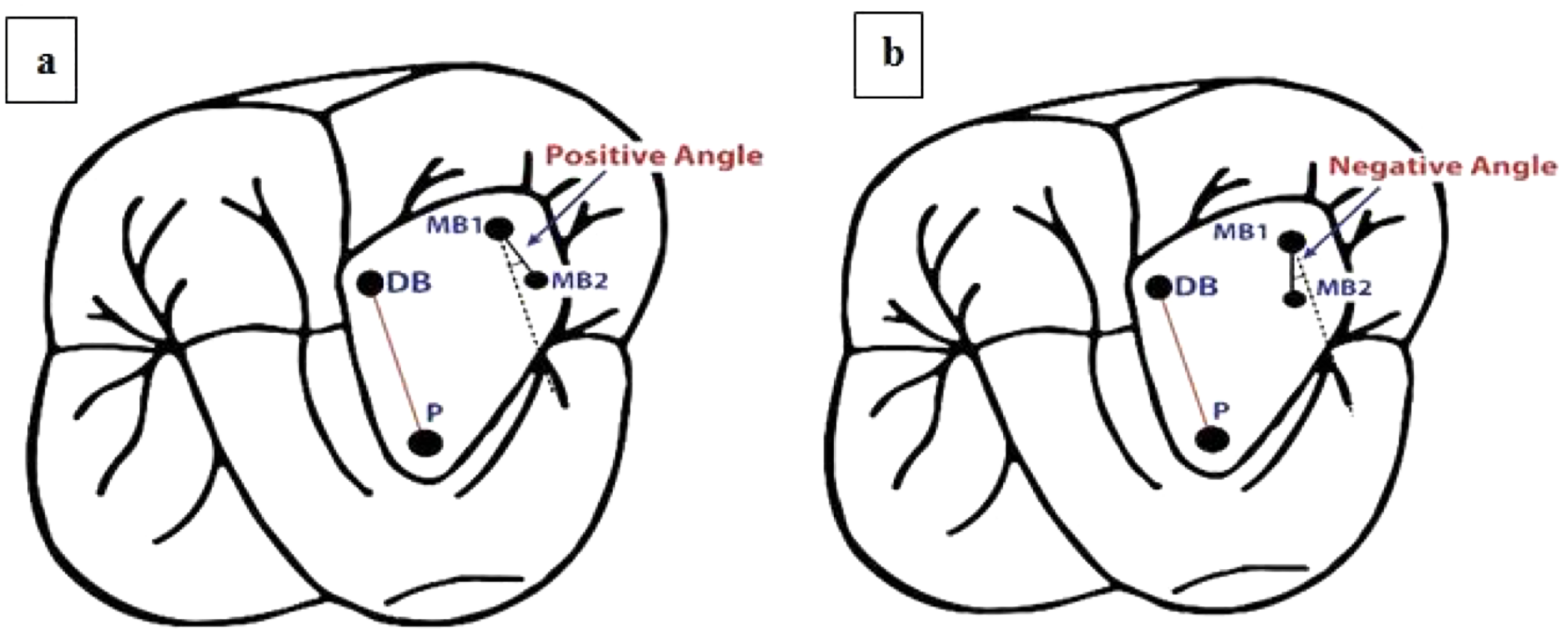
Schematic representation of angular classification for (A) positive angle and (B) negative angle.
  - The angle is described as negative when the MB2 canal is more distal than the equivocal line collateral to the line connecting the DB and P canals passing through MB1 (Fig. 1B).
5. The proposed BVP’s classification has been submitted to the Copyright Office Government of India (8327/2022-CO/L; Dated: 20/04/2022).

## Results

**Figure 2:**
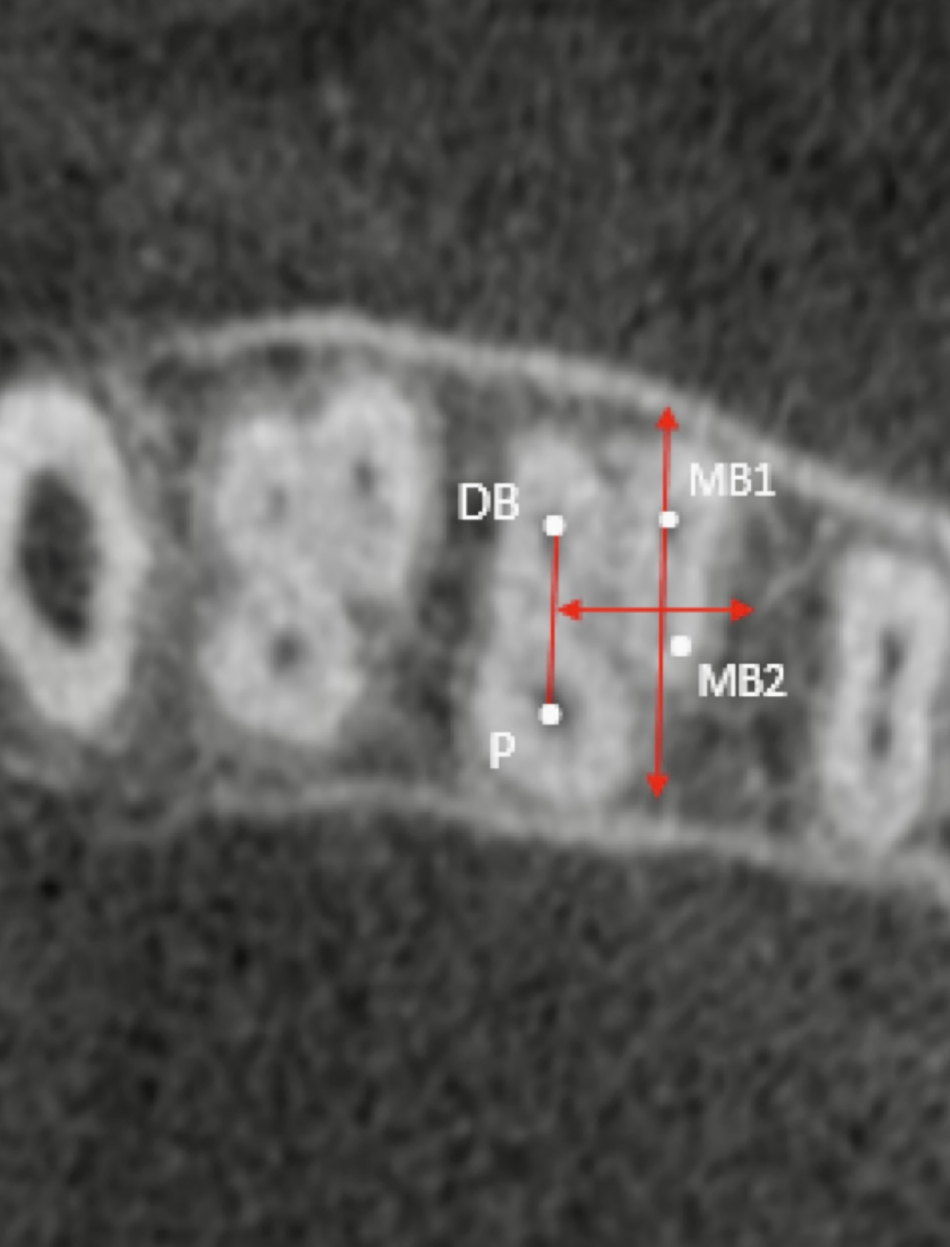
Representative CBCT scan of the evaluated mean distance between the main mesiobuccal and second mesiobuccal canal orifices.

The parametric observation was achieved by marking the x- and y-axes. The y-axis is the imaginary line passing through MB1. This imaginary line is parallel to the line connecting the DB-P canal. The MB1–MB2 mean distance is assumed to equal the highest y-axis value and the zero point (Fig. 2).

${"}{\rm X = (Mb1\cdot Mb2 \, distance) \times sin\alpha} {"} $

${"} \rm Y = Mean \, distance - (Mb1 \cdot Mb2 \, distance) \times cos\alpha " $

### Angle between MB1–MB2 and DB-P canals maxillary first molar

**Figure 3:**
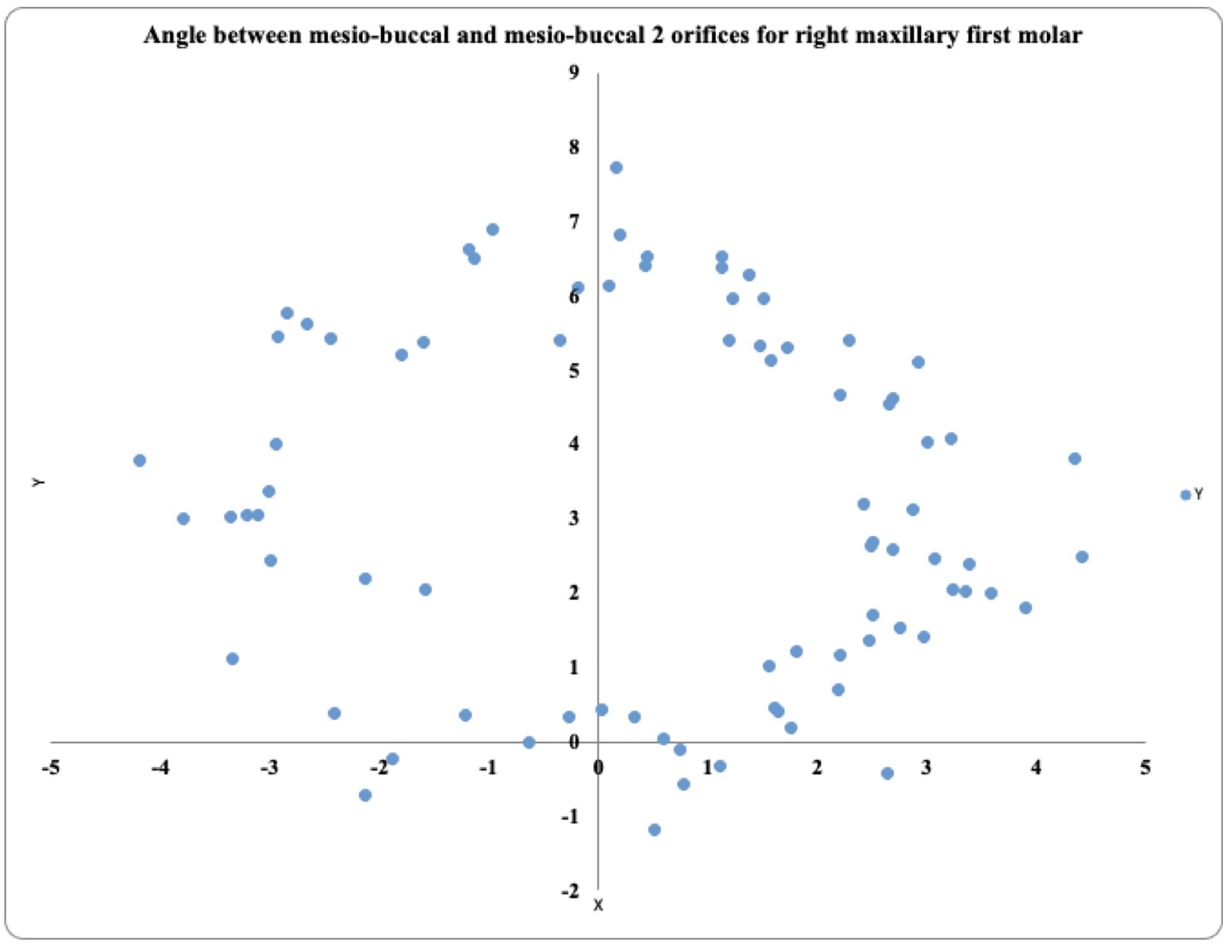
Location angle of the mesiobuccal 2 canal orifice of the maxillary first molar on the right side.

In the 81 maxillary right first molars, the prevalence of MB2 was highest in the positive angle >4° exhibited by 36 teeth, followed by the positive angle 0.1° to 1.9° in 19 teeth and the positive angle 2° to 4° in 17 teeth. These teeth also exhibited negative angles (−2° to −4°) in 5 teeth, (>−4°) in 3 teeth, and (−0.1° to −1.9°) in 1 tooth (Fig. 3).

**Figure 4:**
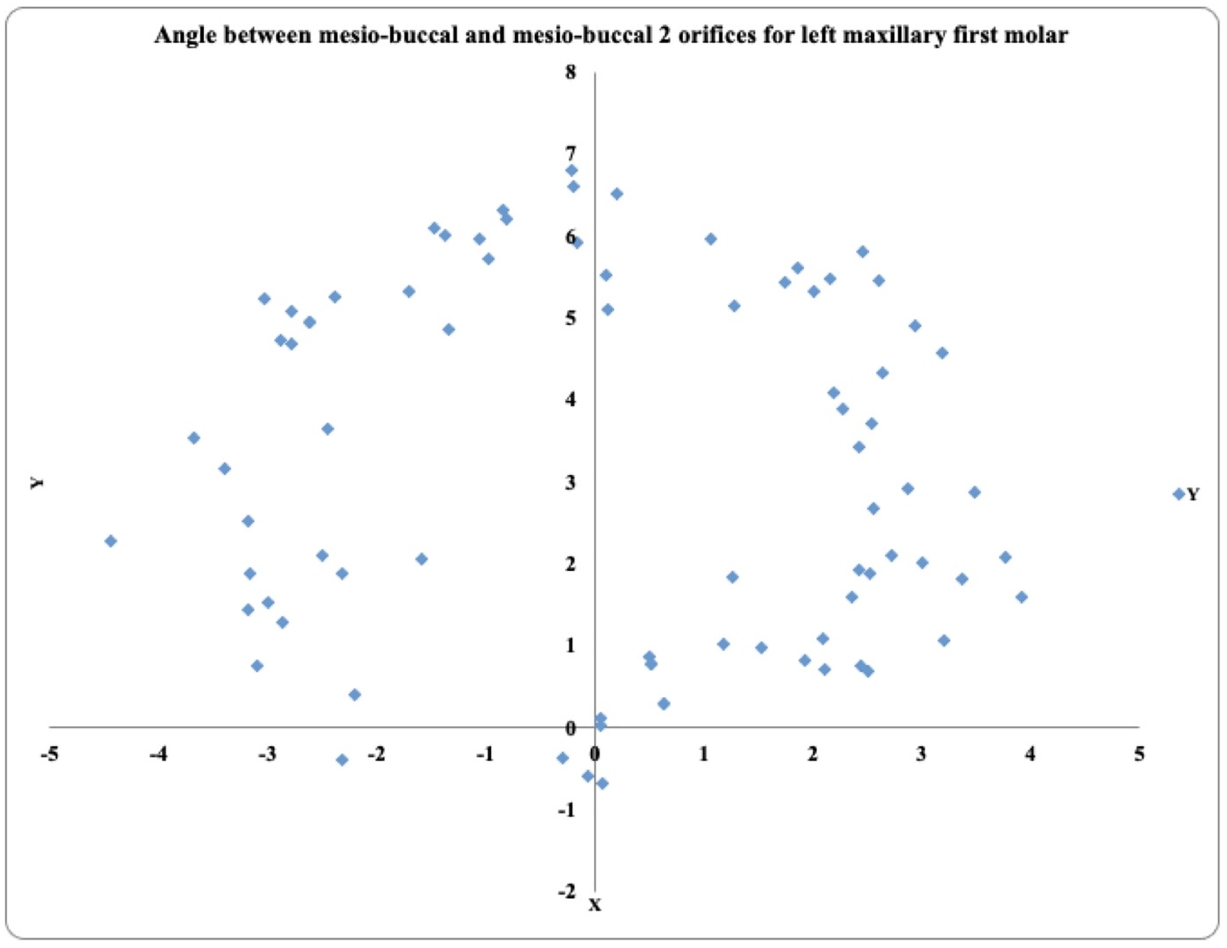
Location angle of the mesiobuccal 2 canal orifice of the maxillary first molar on the left side.

In 81 maxillary left first molars, the prevalence of MB2 was highest in the positive angle >4° exhibited by 31 teeth, followed by the positive angle 2° to 4° in 24 teeth and the positive angle 0.1° to 1.9° in 15 teeth. These teeth also exhibited negative angles (−2° to −4°) in 6 teeth, (>−4°) in 4 teeth, and (−0.1° to −1.9°) in 2 teeth (Fig. 4).

### Angle between MB1–MB2 and DB-P canals maxillary second molar

**Figure 5:**
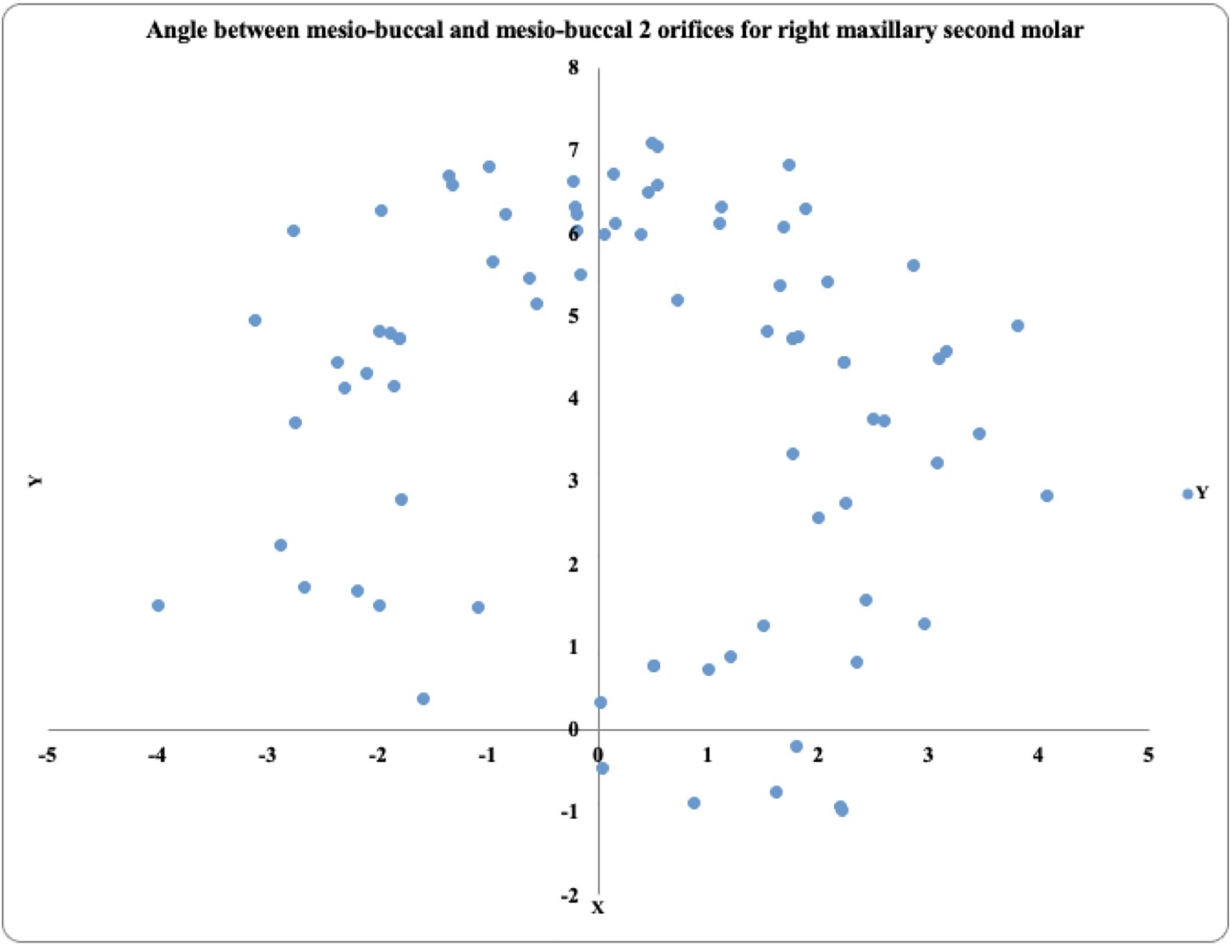
Location angle of the mesiobuccal 2 canal orifice of the maxillary second molar on the right side.

In 81 maxillary right second molars, the MB2 prevalence was highest in the positive angle 2° to 4° exhibited by 32 teeth, followed by the positive angle >4° in 23 teeth and the positive angle 0.1° to 1.9° in 14 teeth. These teeth also exhibited negative angles (−2° to −4°) in 8 teeth, (>−4°) in 4 teeth, and no tooth exibhited (−0.1° to −1.9°) (Fig. 5).

**Figure 6:**
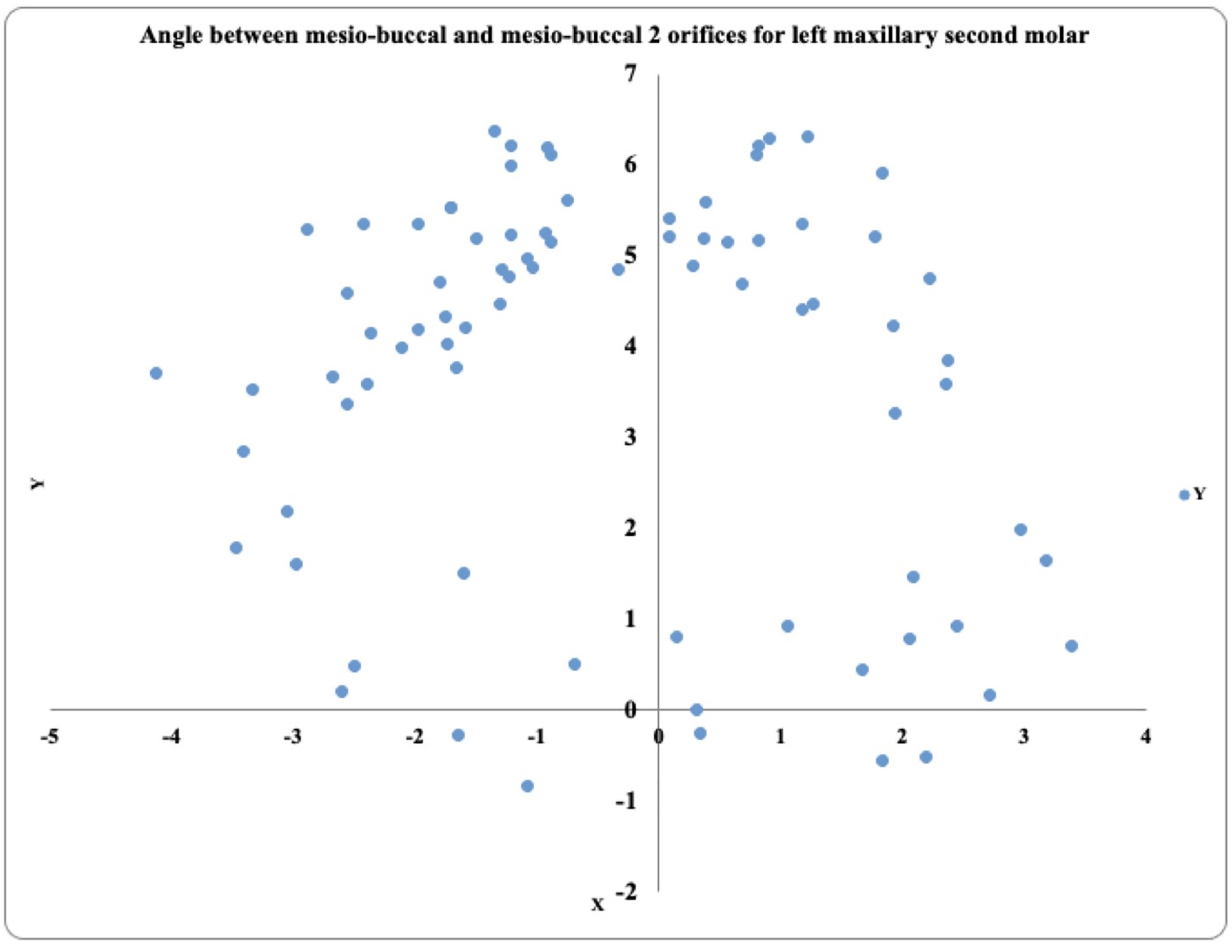
Location angle of the mesiobuccal 2 canal orifice of the maxillary second molar on the left side.

In the 81 maxillary left second molars, the MB2 prevalence was highest in the positive angle 2° to 4° exhibited by 36 teeth, followed by the positive angle >4° in 26 teeth and the positive angle 0.1° to 1.9° in six teeth. These teeth also exhibited negative angles (−2° to −4°) in 8 teeth, (>−4°) in five teeth, and none of the teeth were present (−0.1° to −1.9°) (Fig. 6).

### The MB1–MB2 canal orifice distance

The mean distances between the MB1–MB2 canal orifices were 3.12–3.31 mm in the first molars and 2.8–3.1 mm in the second molars. The *p* value is 0.000, which is highly significant (Table 4).

**Table 4:** Mean distance between the main mesiobuccal and second mesiobuccal canal orifices.

Tooth type	Distances (mm) and standard deviation (±)
Maxillary right 1^st^ molar	3.3 ± 0.55
Maxillary left 1^st^ molar	3.12 ± 0.50
Maxillary right 2^nd^ molar	3.11 ± 0.65
Maxillary left 2^nd^ molar	2.88 ± 0.60

### Distance between DB-P canal orifices

The mean distance between the disto-buccal and palatal canal orifices was 5.06–5.22 mm in the maxillary first molars and 4.9–5.8 mm in the maxillary second molars (Table 5).

**Table 5:** Mean distance between the disto-buccal and palatal canal orifices.

Tooth type	Distances (mm) and standard deviation (±)
Maxillary right 1^st^ molar	5.22 ± 0.78
Maxillary left 1^st^ molar	5.72 ± 0.65
Maxillary right 2^nd^ molar	5.90 ± 0.66
Maxillary left 2^nd^ molar	4.95 ± 0.68

### Location of the MB2 canal at the cementoenamel junction

Most commonly, the location of MB2 is between 0.5 and 1.00 mm below the CEJ. The results are presented in Table 6.

**Table 6:** Level of the location of the mesiobuccal 2 canal with respect to the cementoenamel junction.

MB2 orifice below the level of CEJ	Maxillary 1^st^ molar	Maxillary 2^nd^ molar
0.5 mm	23	31
1 mm	96	90
1.5 mm	37	26
2 mm	6	15

### Application of BVP’s angular classification

**Figure 7:**
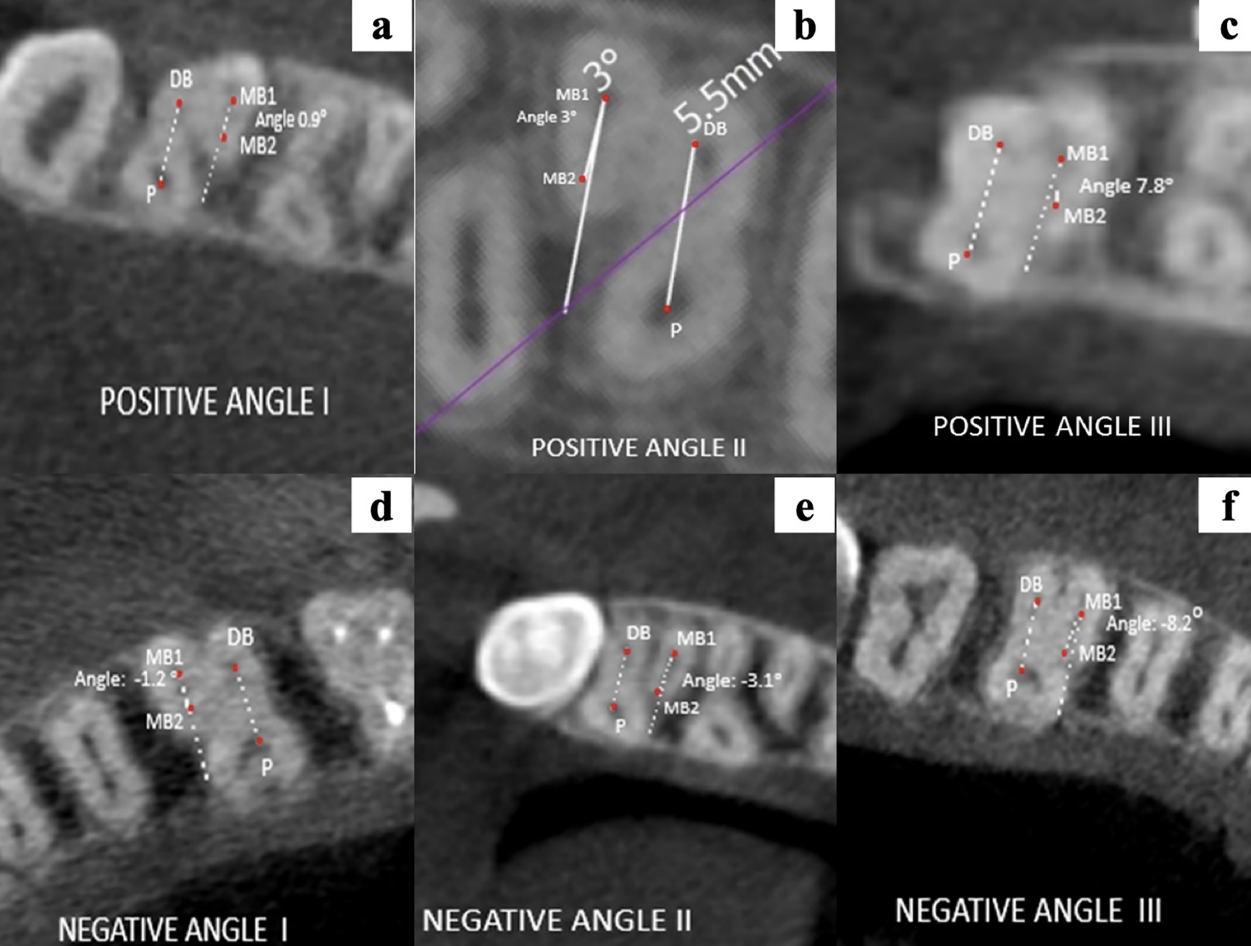
(A–F) Representative images of the different location angles seen in maxillary molars according to BVP’s angular classification.

The location of MB2 after application of BVP’s angular classification is represented in Figs. 7A–7F.

## Discussion

The anatomical morphology of roots and the canal of teeth vary to a high extent. The frequency of missed canals is solely because of morphological variation of canals in the tooth root. It is assumed that there are two or more canals in the MB root (Cantatore, Berutti & Castellucci, 2006).

In the current study, CBCT technology was selected to assess the location of the MB2 canal in maxillary molars. The usage of CBCT for canal detection is a practical method. It is one of the reliable diagnostic methods for detecting and studying anatomical variation presented in an individual (Alfouzan et al., 2019; Martins et al., 2018). In computed tomography, anatomical structures such as teeth and their neighboring structures are seen in three planes (Olczak & Pawlicka, 2017).

CBCT provides quite favorable results and has upgraded the ease and possibility of reading otherwise missed MB2 canals during the procedure. It is advantageous over other diagnostic measures, as it is noninvasive and thin slices permit adequate visualization without distortion of the image (Agwan et al., 2015; Nikoloudaki, Kontogiannis & Kerezoudis, 2015). The teeth excluded in the present study were those with metal posts, root canal treatment or rehabilitation cases with fixed prostheses. These types of subjects were excluded to ensure scatter-free images owing to their higher density nature (Betancourt et al., 2016). Because of the exposure period, the radiation dosage for CBCT is dependent on the voxel resolution. The higher the voxel size is, the greater the acquisition needed; therefore, the radiation exposure increases. CBCT has good sensitivity and specificity for the diagnosis of MB2 (Betancourt et al., 2015). It helps practitioners perform a safe, effective, and predictive endodontic treatment (Stropko, 1999; Hofmann & Thorpe, 2002; Vizzotto et al., 2013).

However, dental professionals should also follow the ALARA principle. The fundamental concepts of radiation dosage exposure justification, optimization, and limiting. The updated and improved CBCT software has enabled upgrading the resolution of images. This leads to greater diagnostic accuracy and precision (Fernandes et al., 2019). The determination of tooth length on CBCT scans was trustworthy and accurate, according to an analysis of dental anatomy using CBCT and conventional periapical radiography (Naseri et al., 2018).

The current study measured the MB1–MB2 canal distance in an Indian population. The presence of the MB2 canal was determined by studying the angles between the imaginary lines. These lines connecting MB1–MB2 and the DB-P canal orifice were considered. The images were rebuilt in this investigation so that the tooth’s long axis was perpendicular to the plane going through the CEJ. The observation plane was positioned 1 mm below the horizontal plane traveling through the pulpal floor. The CEJ was referenced for restructuring the image to allow simple recognizability with highly reproducible results. Clinicians acknowledge the root canal orifice at the pulpal floor after access cavity preparation, which further increases the ease of locating the canal (Lee et al., 2020; Deutsch et al., 2005).

The distance between MB1 and MB2 in the upper 1^st^ molar was 3.12–3.31 mm, while this distance in the maxillary 2^nd^ molar was 2.8–3.1 mm (Table 4). Similar results were seen by Sujith et al. (2014) in his study on the maxillary 1^st^ molar. It was found that the MB2 canal can be located within 5 mm of the MB1 canal (Sujith et al., 2014). The study performed by Lee et al. (2020) on 211 teeth showed that the MB1–MB2 canal distance between the orifices of the first molar was 2.53 mm for the adjacent molar, it was 2.42 mm, both in the unknown population. A similar observation was found in a study with 2.67 mm and 2 mm in maxillary first and second molars (1,100 teeth), respectively (Betancourt et al., 2016). It has been shown that detection of the MB2 canal was more successful if channeling was performed with the help of an ultrasonic tip in the chamber floor, particularly within 3 mm from the MB canal toward the palatal canal (Wolcott et al., 2002).

The present study places more emphasis on the actual location of the second mesiobuccal canal rather than its incidence. The angle studied connecting the lines MB1–MB2 and DB-P canals defines the position of MB2. The angle was read as positive if the MB2 canal was more mesial than the imaginary line parallel to the line linking the DB and P canals going through MB1. When the MB2 canal was further away from the imaginary line, however, the interpretation was negative.

Sixty-seven maxillary first molars showed positive angle III, and sixty-eight maxillary second molars showed positive angle II. Another study was performed by Lee et al. (2020), in which the angles obtained were 2.30° ± 5.70° in the first molar and −3.95° ± 7.73° in the second. The angles between the connecting imaginary lines were within ±8°. Another finding stated that the above mentioned angle was strictly mesial in the first two molars. No such study has been performed by Indian authors thus far.

During endodontic procedures on maxillary molars, clinical practitioners should acquire adequate knowledge of tooth morphology. From the inferences obtained from the study, it can be stated that the most frequent position of MB2 is within 3 to 3.5 mm of MB1. This finding will facilitate easy locating of otherwise missed MB2 canals to ensure adequate success in endodontic procedures for upper molars.

## Conclusions

Accurate diagnosis of the location of the second mesiobuccal canal increases the success rate of endodontic treatment and leads to a better prognosis. The classification proposed in the present study further aids in detecting the precise location of MB2 with respect to measurements of distance and angle.

## Supplemental Information

10.7717/peerj.14234/supp-1 Supplemental Information 1 Raw data.

## Additional Information and Declarations

### Data Availability

The following information was supplied regarding data availability: The raw measurements are available as a Supplemental File.
